# Investigation of the Molecular Mechanisms of the Eukaryotic Cytochrome-*c* Maturation System

**DOI:** 10.3390/biom12040549

**Published:** 2022-04-07

**Authors:** Ana V. Silva, Maria O. Firmino, Nazua L. Costa, Ricardo O. Louro, Catarina M. Paquete

**Affiliations:** Instituto de Tecnologia Química e Biológica António Xavier, Universidade Nova de Lisboa, Av. da República, 2780-157 Oeiras, Portugal; anavsilva@itqb.unl.pt (A.V.S.); mfirmino@itqb.unl.pt (M.O.F.); nazuacosta@itqb.unl.pt (N.L.C.); louro@itqb.unl.pt (R.O.L.)

**Keywords:** lyase, multiheme cytochrome, Small Tetraheme Cytochrome, CcHL

## Abstract

Cytochromes-*c* are ubiquitous heme proteins with enormous impact at the cellular level, being key players in metabolic processes such as electron transfer chains and apoptosis. The assembly of these proteins requires maturation systems that catalyse the formation of the covalent thioether bond between two cysteine residues and the vinyl groups of the heme. System III is the maturation system present in Eukaryotes, designated CcHL or HCCS. This System requires a specific amino acid sequence in the apocytochrome to be recognized as a substrate and for heme insertion. To explore the recognition mechanisms of CcHL, the bacterial tetraheme cytochrome STC from *Shewanella oneidensis* MR-1, which is not a native substrate for System III, was mutated to be identified as a substrate. The results obtained show that it is possible to convert a bacterial cytochrome as a substrate by CcHL, but the presence of the recognition sequence is not the only factor that induces the maturation of a holocytochrome by System III. The location of this sequence in the polypeptide also plays a role in the maturation of the *c*-type cytochrome. Furthermore, CcHL appears to be able to catalyse the binding of only one heme per polypeptide chain, being unable to assemble multiheme cytochromes *c*, in contrast with bacterial maturation systems.

## 1. Introduction

Cytochromes-*c* are essential proteins for living organisms across all domains of life, serving as an essential component of several metabolic processes such as respiration [1,2], apoptosis [3,4], and extracellular electron transfer [5,6]. This class of proteins is defined by the stereo-specific covalent attachment of the heme cofactor to the polypeptide chain via two (rarely one) thioether bonds to cysteine side chains [2,7]. This allows for the unique feature of binding a virtually limitless amount of heme cofactors per polypeptide chain [5]. For this covalent attachment to occur, a dedicated maturation biochemical machinery is required [8]. Although three main cytochrome-*c* maturation systems have been described [9], together with variations that constitute subsequent numbering [10,11,12], the most prevalent in nature are Systems I and III. System I is constituted of up to nine membrane-bound components (for a review, see [13]), and it is present in α-and γ-proteobacteria, in Archaea, and in the mitochondria of plants and red algae. This system is able to mature all classes of cytochromes-*c*, including multiheme cytochromes [9,13]. In contrast, System III involves only one essential protein, designated cytochrome-*c* heme lyase (CcHL) [14] or holocytochrome *c* synthetase (HCCS) [15]. System III is present in the mitochondria of most eukaryotic organisms, with the exception of plants [16], being, so far, only found in organisms that do not produce multiheme cytochromes. In this work, for historical reasons, we will use the original name of the enzyme, CcHL [17]. CcHL is predicted to be a soluble membrane-associated protein with 170 to 289 residues [16]. In contrast to Systems I and II, System III requires not only the presence of the CX_n_CH heme-binding motif but also of a consensus recognition sequence that is different for different organisms and that has been identified for both humans and yeast [14,18]. It has been demonstrated that this sequence is responsible for the formation of an alpha helix required for CcHL to recognize and attach its substrate [19].

**Figure 1:**
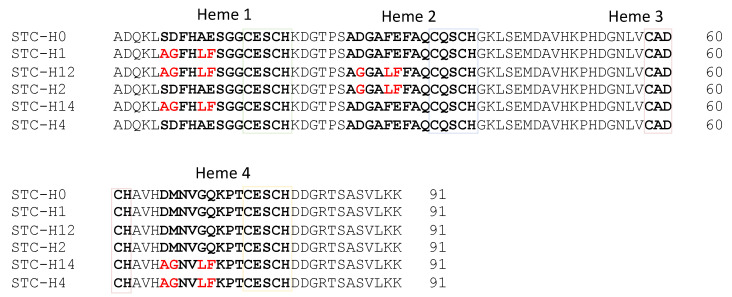
Amino acid sequence of the different STC mutants with mutated residues highlighted in red. Amino acids in bold highlight the recognition sequence and heme-binding motif of the hemes that were mutated.

In humans, the malfunction of System III has several health implications, being the sole cause of the genetic disease microphthalmia with linear skin defects (MLS) [20]. This syndrome is lethal in males and leads to several complications in females, including mental retardation [21]. Furthermore, System III has also been implicated in a caspase-independent cell death pathway in injured neurons [22], although the mechanisms by which CcHL triggers apoptosis are still to be identified. Despite the acknowledged importance of System III in human health, this is the least understood maturation system as the enzyme was never fully characterized, and its recognition and maturation mechanisms remain incompletely understood. To address the recognition mechanisms of CcHL, in this work, we tested the ability of the yeast heme lyase to produce a class III multiheme cytochrome, the Small Tetraheme Cytochrome (STC) from *Shewanella oneidensis* MR-1. STC is a fully characterized protein of similar size to the mitochondrial cytochrome-*c*, being involved in the extracellular electron transfer pathway of *S. oneidensis* MR-1 [23,24]. STC has four CXXCH heme-binding sites, and mutations were made to implement the known recognition sequence for CcHL before the various heme-binding sites (Figure 1).

Here, we demonstrate that CcHL is able to insert only one heme in a multiheme apo-cytochrome and that the distance between the N-terminal and the recognition sequence influences heme attachment. Based on these results, the four-step model of the CcHL-mediated cytochrome *c* biogenesis, previously proposed by Babbit et al. [16], is updated.

## 2. Materials and Methods

### 2.1. Production of STC Mutants with System III

To study System III, we used an expression system that produces the enzyme for the maturation of yeast, horse and human cytochromes-*c* in *E. coli* [25,26,27,28]. To produce native STC (hereafter simply referred to as STC) using this system, the *cctA* gene from *S. oneidensis* MR-1, encoding the tetraheme cytochrome STC without its native signal peptide, was cloned in the plasmid pBTR-1 that contains the *S. cerevisiae* CcHL gene [25], using the primers presented in Table S1. To facilitate the purification procedure, the construct was built to express a 6× Histidine-tag at the C-terminus of the protein. The resulting plasmid was denominated pCPH0. Mutations to insert the consensus sequence for CcHL recognition in the binding region of heme 1 were introduced by site-directed mutagenesis using the primers indicated in Table S1 and using the manufacturers’ procedure (NZYTech, Lisboa, Portugal), resulting in the plasmid pCPH1. The same procedure was used to build the plasmids pCPH2 and pCPH4 that contain the consensus CcHL recognition sequence in the binding site for hemes 2 and 4, respectively. Finally, site-directed mutagenesis was performed on pCPH1 to build the plasmids pCPH12 and pCPH14 that contain the consensus sequence inserted in hemes 1 and 2, and 1 and 4, respectively. All constructs were confirmed by DNA sequencing (Eurofins Genomics, Ebersberg, Germany). The resulting plasmids were then transformed in *E. coli* BL21 (DE3) cells. Given that the mutations in the third heme-binding position would affect the second histidine axial ligand of this heme (see Figure 1), this experiment was not performed for mutant STC-H13.

Pre inocula were initiated from a single freshly transformed colony and grown overnight at 37 °C with shaking at 150 rpm in 100 mL of Luria–Bertani medium (LB) [29] supplemented with 100 µg/mL ampicillin. For protein expression, cultures were diluted to an OD_600_ of 0.1 in Terrific Broth medium (TB) [30] with 100 µg/mL ampicillin and grown aerobically at 37 °C for 48 h with shaking at 150 rpm. Cells were harvested by centrifugation at 10,000× *g* at 4 °C for 10 min and frozen at −20 °C.

For protein purification, cells were defrosted and resuspended in 20 mM Tris-HCl buffer at pH 7.6, supplemented with protease inhibitor cocktail (Roche, Basel, Switzerland) and DNase I (Sigma-Aldrich, St. Louis, MO, USA). Cell disruption was performed using a French Press by passing the cells three times at 1000 psi. The lysed cells were ultracentrifuged at 210,000× *g* at 4 °C for 90 min, and the resulting soluble protein extract was purified using a 5 mL pre-packed His-trap column (GE Healthcare, Chicago, IL, USA), previously equilibrated with 20 mM potassium phosphate buffer at pH 7.6 with 300 mM NaCl buffer. A linear gradient from 0 to 100% buffer B (20 mM potassium phosphate buffer at pH 7.6 with 300 mM NaCl and 500 mM Imidazole) in 150 mL was used to elute the target protein. All chromatographic fractions were analyzed in 13% acrylamide SDS-PAGE gels. The gels were stained by both heme stain and BlueSafe (NZYTech, Lisboa, Portugal). The heme stain protocol, which is based on the heme peroxide activity, allows the produced *c*-type cytochrome to be identified on an SDS gel electrophoresis [31]. Fractions containing the target protein were concentrated using centrifugal filter units (Millipore, Burlington, MA, USA) with a 5 kDa cut-off membrane. Pure protein was revealed as a single band in the BlueSafe-stained SDS-PAGE gel (Figure S1). The same purification procedure was used for all the mutants.

### 2.2. Production of STC Mutants with System I

The production of STC using System I by *E. coli* was performed as previously described [23]. Mutations to introduce the consensus sequence for CcHL recognition in the heme-binding sites of heme 1, 2, and 4, to produce STC-H1, STC-H2, and STC-H4, were performed with site-directed mutagenesis using the manufacturers’ procedure (NZYTech, Lisboa, Portugal) using the primers listed in Table S1. Additional site-directed mutagenesis was performed on STC-H1 to build the plasmids STC-H12 and STC-H14, adding the consensus sequence for hemes 2 and 4, respectively. The resulting plasmid was transformed in *E. coli* BL21 (DE3) containing the plasmid pEC86, responsible for the expression of Ccm proteins under aerobic conditions [32]. All constructs were confirmed by DNA sequencing (Eurofins Genomics, Ebersberg, Germany).

Pre inocula were initiated from a single freshly transformed colony and grown overnight at 37 °C with shaking at 150 rpm in 100 mL of LB medium with 100 µg/mL ampicillin and 34 µg/mL chloramphenicol. For protein expression, starter cultures were diluted to an OD_600_ of 0.1 in TB medium with 100 µg/mL ampicillin and 34 µg/mL chloramphenicol and left to grow aerobically at 37 °C for 48 h with shaking at 150 rpm. Cells were then harvested by centrifugation at 10,000× *g* at 4 °C for 10 min and frozen at −20 °C. Disruption was performed by osmotic shock using a solution containing 20 mM Tris–HCl pH 8.0, 20% sucrose, 1 g/L of lysozyme, 0.5 mM EDTA, protease inhibitor cocktail (Roche, Basel, Switzerland), and DNase I (Sigma-Aldrich, St. Louis, MO, USA) according to the previously described protocol [23]. The lysate was incubated with shaking for 90 min at 4 °C.

For protein purification, the lysed cells were centrifuged at 210,000× *g* at 4 °C for 90 min, and the resulting soluble protein extract was purified using a Q-Sepharose ^TM^ Fast Flow Column (GE Healthcare, Chicago, IL, USA) previously equilibrated with 20 mM Tris buffer at pH 7.6. The target protein was eluted at 20 mM of NaCl in the same buffer. When necessary, a second purification step was performed using a Superdex 75 10/300 GL size exclusion column (GE Healthcare, Chicago, IL, USA). All chromatographic fractions were analyzed in 13% acrylamide SDS-PAGE gels. The gels were stained both by heme stain [31] and BlueSafe (NZYTech, Lisboa, Portugal). Pure protein was revealed as a single band in the BlueSafe-stained SDS-PAGE gel. The same purification process was used for all the mutants.

### 2.3. Protein Analysis

The identity of the produced proteins was confirmed by N-terminal sequencing (first five amino acid residues) using the Edman degradation method in an ABI Procise Protein Sequencer, an ABI Microgradient Pump System and an ABI Programmable Absorbance Detector.

UV-visible spectra were recorded at room temperature in a Shimadzu UV-1800 spectrophotometer in the range of 250–800 nM. Data were analysed in the UV-Probe v.2.52 software and MS Excel.

Mass spectrometry analysis was performed in the proteins produced with System III to confirm the number of hemes in the protein. The proteins were applied directly onto the MALDI plate with 1 μL of 5 mg/mL CHCA (alpha-cyano-4-hydroxycinnamic acid, Sigma-Aldrich, St. Louis, MO, USA) in 50% (*v*/*v*) acetonitrile and 5% (*v*/*v*) formic acid. The data were acquired in Linear Low Mass Positive mode using a 4800plus MALDI-TOF/TOF (ABSciex) mass spectrometer and 4000 Series Explorer Software v.3.5.3 (Applied Biosystems) available in the Mass Spectrometry Unit (UniMS), ITQB/iBET, Oeiras, Portugal. External calibration was performed using CalMix5 (Protea).

## 3. Results

Native STC from *S. oneidensis* MR-1 was co-expressed in *E. coli* with yeast CcHL using the pCPH0 plasmid containing both the cytochrome and CcHL genes (construct STC-H0). In this construct, the signal peptide of the STC gene, required for the transport to the periplasm and subsequent maturation by System I, was removed to guarantee that the apo-protein would be maturated by System III in the cytoplasm of *E. coli*. During protein production, aerobic conditions were ensured by shaking the culture at 150 rpm to avoid the expression of the native cytochrome maturation system (i.e., System I) [33]. By performing an SDS-PAGE gel with the cellular fraction of the *E. coli* culture harboring construct STC-H0, no heme stained band could be observed around 12 kDa (i.e., MW of native STC), (Lane 1 in Figure S2), indicating that STC-H0 is not maturated in *E. coli*. This observation is in agreement with the fact that this construct does not contain the consensus recognition sequence required for the maturation process by CcHL [14]. The insertion of the consensus recognition sequence in the first heme-binding position (mutant STC-H1) led to the production of a cytochrome-*c* that is visible by heme staining at approximately 10 kDa (Lane 2 in Figure S2).

**Figure 2:**
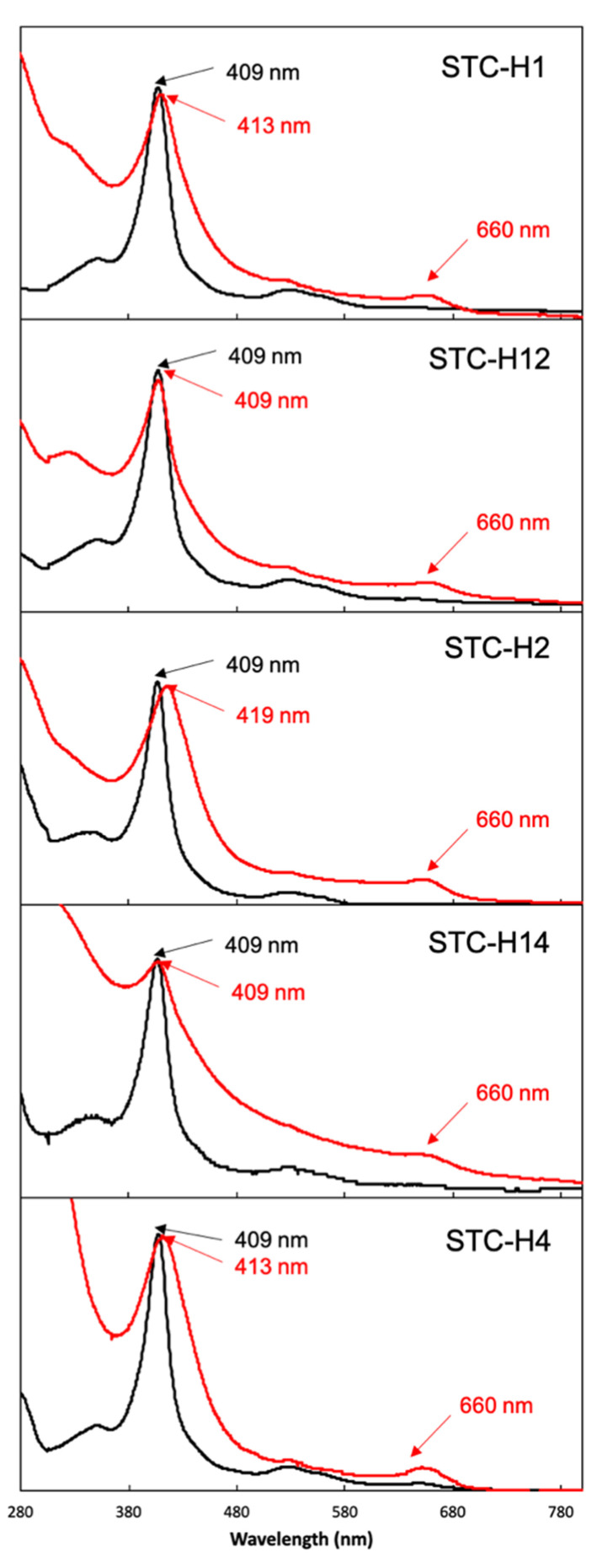
UV-visible spectra of STC mutants produced by System III (red) and System I (black).

The protein produced by CcHL was purified (Figure S1). It displays characteristic features of a high-spin cytochrome-*c*, with a broad Soret peak and the presence of a peak at approximately 660 nm (Figure 2). This suggests that the heme(s) in the protein produced by this maturation system either lack the distal axial ligand or this has been replaced by a weak-field ligand (e.g., water). Indeed, mass spectrometry revealed that only one heme was inserted in STC-H1 (Table 1). The lack of the other three hemes offers a rational explanation for the incomplete folding of the protein and lack of coordination of the heme by the distal axial histidine. These results indicate that the recognition sequence prior to the first heme binding position of STC is required for the polypeptide to be recognized as an apocytochrome by CcHL. However, it is not sufficient for the insertion of all four hemes.

To investigate if CcHL is able to insert more than one heme, STC was mutated to contain the consensus recognition sequence prior to the first and second heme-binding positions (mutant STC-H12). This protein was successfully produced in *E. coli* by System III (Lane 3 in Figure S2) and purified (Figure S1). The position of the band of this mutant in the gel is, however, slightly higher than that of mutant STC-H1. This, together with the fact that the UV-visible spectrum is also different from that of STC-H1 (Figure 2), suggests that the produced protein is different from STC-H1. Mass spectrometry revealed that a mixture of the apo-protein (i.e., protein without the insertion of any heme, of ~10.4 kDa) and protein containing one heme (~11 kDa) exists, being the contribution of both forms very similar (Table 1 and Figure S3). This may explain the different UV-visible spectra and indicates that CcHL was able to insert one heme in this construct but that it was less efficient than insertion in STC-H1.

To investigate why CcHL was not able to insert more than one heme, three different hypotheses were explored: (i) the second consensus sequence is too far from the N-terminus of the protein to be recognized by CcHL; (ii) the distance between the two binding positions is too short, preventing the insertion of the second heme (i.e., the local folding upon the insertion of the first heme impairs the recognition of the second consensus sequence by CcHL), or; (iii) the CcHL can only insert one heme, regardless of the number of consensus sequences in the apo-cytochrome.

To test hypothesis (i), the STC-H2 mutant (insertion of the recognition sequence in the second heme-binding position) was constructed. This protein was successfully produced by CcHL (Figure S1 and Lane 4 in Figure S2). However, the position of the band of this mutant in the gel (Lane 4 in Figure S2) is different from the other mutants, showing that the insertion of the heme in this position affected the electrophoretic mobility of the protein in the SDS-PAGE. The UV-visible spectrum corroborates the different properties of this mutant, which displays a shift in the Soret peak from 409 nm to 419 nm (Figure 2). Mass spectrometry shows a larger contribution of the apo-form of the protein (~10.4 kDa) versus the form containing one heme (~11 kDa) (Table 1 and Figure S3). These results show that heme binding occurs and that the distance of the recognition sequence from the N-terminus impacts the efficiency of heme insertion by CcHL. These results eliminate hypothesis (i).

To test hypothesis (ii), mutant STC-H14 (insertion of the recognition sequence in the first and fourth heme-binding positions) was built and produced (Figure S1 and Lane 5 in Figure S2). While the position in the SDS-PAGE gel (Lane 5 in Figure S2) and UV-visible spectrum of this mutant was similar to that observed for the STC-H12 mutant (Figure 2), mass spectrometry results showed a larger population of the apo-form of the protein (~10.4 kDa) versus the form with one heme inserted (~11 kDa) (Table 1 and Figure S3). For STC-H14, only a small population of the protein form that contains one heme could be observed, with no evidence of protein containing more than one heme. This reveals that the proximity between the two heme-binding positions was not the reason for the inability of CcHL to place more than one heme, discarding hypothesis (ii).

To investigate if it is the distance from the N-terminus to the last heme-binding position that impairs the second heme insertion in mutant STC-H14, the mutant STC-H4 (insertion of the recognition sequence in the fourth heme-binding position) was built and produced by CcHL (Lane 6 in Figure S2). The UV-visible spectrum of this mutant is, however, different when compared with the other mutants (Figure 2), including a modification in the Soret peak shape and relative intensity when compared to the 280 peak. Although this protein presents the characteristic properties of a cytochrome-*c*, namely a broad Soret peak at 413 nm in the UV-visible spectrum, and a band that stains for hemes in the SDS-PAGE gel (Lane 6 in Figure S2), the mass spectrometry signal is dominated by the apo-form of the protein (Table 1). This is in agreement with the high intensity of the 280 nm peak and suggests that the heme-containing population is of very low abundance and does not emerge from the envelope of mass spectrometry signal. These results indicate that the distance of the consensus recognition sequence to the N-terminal is important for the efficiency of CcHL in maturating cytochromes-*c*.

It has to be mentioned that all the target proteins have been properly assembled by its native cytochrome maturation system (i.e., System I) (Figure 2). This clearly shows that the lack of insertion of more than one heme is not because of the mutations in the consensus sequence but due to the mode of action of CcHL.

## 4. Discussion

Cytochrome-*c* maturation System III has been studied since the 1970s [34], and in 1987, the gene responsible for the process was identified in yeast [17]. The study of this system has been extremely challenging, mainly due to the fact that this system is composed of only one protein and low amounts of the enzyme present in cells, preventing its purification [35]. The construct that allowed the purification of this enzyme for the first time was engineered in 2013 when human CcHL was purified from *E. coli* with an N-terminal GST fusion [18]. This was fundamental in advancing the understanding of this system, permitting the identification of key residues in CcHL for heme and apo-cytochrome binding, including the consensus recognition sequence for human CcHL [18]. At the same time, the recognition sequence for yeast CcHL was also identified, demonstrating that the recognition sequences are distinct in different organisms [14].

**Figure 3:**
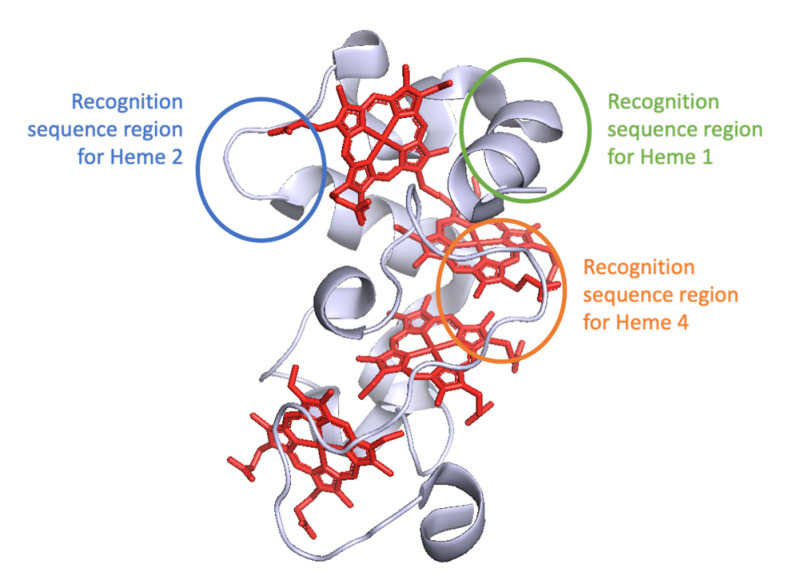
Cartoon representation of STC structure (PDB: 1M1Q) with mutated regions highlighted with colored circles.

In this study, five genetically engineered variants of the tetraheme cytochrome STC from *S. oneidensis* MR-1 were expressed, purified and characterized to understand the mechanisms by which CcHL recognizes a polypeptide as an apo-cytochrome that should undergo maturation into a cytochrome-*c*. These mutations incorporate the recognition sequence for yeast CcHL located prior to the different heme-binding positions in STC (Figure 3). The different variants were produced by both System I and III, to ensure that the differences observed were only due to the maturation system and not to alterations in the polypeptide chain that would affect the protein structure.

The production of STC-H0 was performed as a proof of concept, demonstrating that without the recognition sequence, the apo-protein is not recognized by System III and, consequently, no heme cofactor is introduced in the protein, as previously observed [14]. This is in contrast to what has been observed for System I and II, where a specific consensus recognition sequence is not required for cytochrome-*c* maturation, and only the heme-binding motif CXXCH is required for recognition and heme binding [18,36]. The consensus recognition sequence is part of the alpha helix required for CcHL to recognize the apo-cytochrome and insert the heme [37]. In the case of STC, it was crucial for the insertion of one heme, but not sufficient to place all of the four hemes. The production of both STC-H12 and STC-H14 demonstrated that the inability of CcHL to place more than one heme was not due to the short distance (15 amino acids) that separates the binding sequence of the two heme-binding sites in STC-H12. The fact that these mutants present similar features between them (i.e., run similarly in the SDS-PAGE gel and present comparable spectroscopic features) that are different from the cytochromes with only one consensus sequence (STC-H1, STC-H2 and STC-H4), suggests that the produced proteins in STC-H12 and STC-H14 are a mixture of both forms (i.e., heme bound in positions 1 and 2 in STC-H12, and in positions 1 and 4 in STC-H14). These results show that CcHL recognizes the alpha helix, and after binding the heme, the mature cytochrome-*c* is released, preventing it from inserting more than one heme in a single polypeptide chain. Furthermore, the efficiency of recognition and binding of the heme to the apo-cytochrome is inversely related to the distance of the position of the recognition sequence to the N-terminus of the polypeptide. This hypothesis correlates well with the amount of mature cytochrome-*c* that is obtained for the mutants STC-H1, STC-H2 and STC-H4, where the fraction of apo-protein in the produced protein increases with the distance of the consensus recognition sequence to the N-terminus.

**Figure 4:**
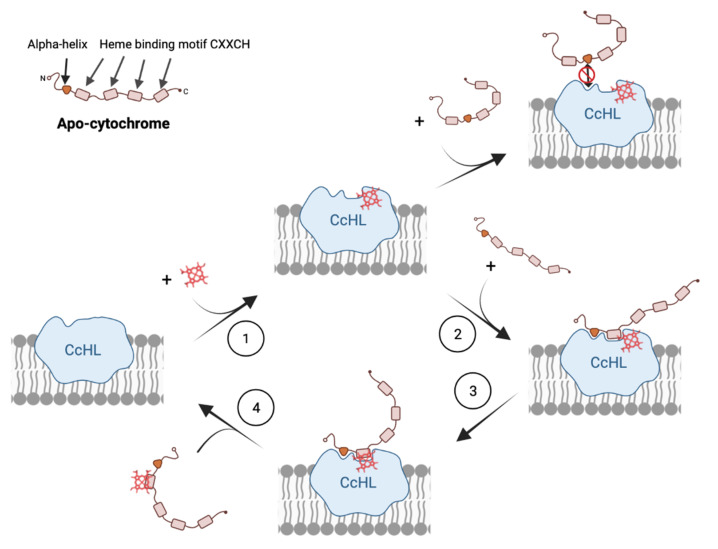
Model for the molecular mechanism of cytochrome-*c* maturation by System III.

The results obtained with this work complement the four-step model previously proposed for the catalytic mechanism of System III [16], adding unique information to steps 2 and 4, where the recognition of the apo-protein by the CcHL and release of the mature protein occur, respectively (Figure 4). Our work demonstrates that the distance of the recognition sequence, and consequently the alpha helix recognized by CcHL to the N-terminus of the apo-cytochrome, plays a key role in the recognition process by CcHL and binding of the heme. When this recognized alpha helix is far from the N-terminus of the apo-protein, the polypeptide chain that precedes it likely causes steric clashes with the CcHL regions surrounding the docking site, disturbing recognition by CcHL and preventing heme binding (see Figure 3). Furthermore, once the heme is bound, the CcHL releases the cytochrome, being unable to identify further recognition sequences.

Therefore, the catalytic mechanisms of cytochrome maturation System III comprise: the binding of the heme by CcHL (step 1), enabling CcHL to be ready to recognize and bind the alpha-helix of a polypeptide without stable secondary or tertiary structural elements (step 2). In this step, it is crucial that the consensus sequence in the apo-protein is easily accessible to be recognized by CcHL, to allow the binding of the heme-binding motif. After binding, CcHL recognizes the heme-binding motif and promotes the attachment of the heme to the apo-cytochrome (step 3), allowing the protein to fold. This enables the mature cytochrome-*c* to be released from CcHL (step 4).

This process is quite distinct from System I and II, where several hemes are bound to a single polypeptide chain, and therefore, several binding motifs have to be identified, probably sequentially in the apo-protein, and more than one heme has to be inserted. The recent cryo-EM structure of CcsBA from the System II cytochrome *c* maturation system revealed that this complex changes from an open to a closed conformation, providing the necessary reaction chamber for the stereochemical attachment of the external heme to the CXXCH heme-binding motif in the apo-cytochrome [36]. Although the tryptophan-rich loop forming the WWD domain, required to provide assistance to heme binding, attachment and release of the cytochrome, was shown to be conserved among the different maturation systems [16,36], structural analysis of the different active sites are quite distinct (Figure S4). This may explain why System III cannot insert more than one heme, while System I and II are able to mature multiheme cytochromes-*c*. In addition to the WWD domain, two conserved periplasmic histidines flanking the WWD domain were shown to be crucial for heme attachment in both Systems I and II [19]. These histidines are absent in CcHL, highlighting the difference between these Systems and CcHL, and may be crucial for the insertion of more than one heme in both Systems I and II. Indeed, the loops around the WWD domain in both the CcsAB complex from System II and CcmC from System I [38] may be important not only for heme motif recognition but also to help in the binding of apo-protein, even after the insertion of a heme and detachment from the active site. This would allow, for example, CcsAB to switch back from the close to an open state with another heme in the external position, necessary to be inserted in the subsequent heme-binding motif of a multiheme cytochrome-*c*. In the case of CcHL, once the heme is bound to the apo-protein, the cytochrome is released from the enzyme, becoming ready to uptake another heme and apo-cytochrome (step four in Figure 4).

## 5. Conclusions

In conclusion, the results obtained with this work unequivocally show that handling of the apo-cytochrome *c* polypeptide chain relies on a different mechanism in System III versus System I and II: System III requires the recognition sequence and the heme-binding motif (i.e., CXXCH) to insert a heme in the apo-cytochrome; the distance between the N-terminal of the apo-protein and the recognition sequence influences heme binding in System III; CcHL can only insert one heme in the apo-protein. This information is relevant to the understanding of how CcHL recognizes its substrates and in finding strategies to manipulate its activity for therapeutic interventions. Further research, including the determination of CcHL structure, and the interaction with its heme and peptide substrates, will be fundamental to fully understand its maturation mechanisms.

## Figures and Tables

**Table 1:** Expected molecular weight (kDa) for the different STC mutants, determined from the molecular mass of the total number of residues plus the molecular mass corresponding the heme group(s) (616.5 Da per heme). Grey shading represents the species detected by mass spectrometry.

Mutant	Apo Protein	1 Heme	2 Hemes	3 Hemes	4 Hemes
STC-H1	10.5	11.1	11.7	12.3	12.9
STC-H12	10.4	11.0	11.6	12.2	12.9
STC-H2	10.4	11.0	11.6	12.3	12.9
STC-H14	10.4	11.0	11.7	12.3	12.9
STC-H4	10.4	11.1	11.7	12.3	12.9

## Data Availability

Not applicable.

## Supplementary Materials

The following supporting information can be downloaded at: https://www.mdpi.com/article/10.3390/biom12040549/s1, Figure S1: Bluesafe SDS-PAGE gel of STC mutants produced by System III; Figure S2: Heme-stained SDS-PAGE gel of STC mutants produced by System III; Figure S3: Mass spectrometry of the STC mutants produced by System III in *E. coli*; Figure S4: WWD domain in the cytochrome maturation Systems; Table S1: List of primers used in the study.
