# Supplementary material for: Investigation of the Molecular Mechanisms of the Eukaryotic Cytochrome-c Maturation System
Source: Biomolecules. 2022 Apr 7;12(4):549. doi: 10.3390/biom12040549 (PMC9028165; doi:10.3390/biom12040549)
Supplement: Supplementary file 1 [file biomolecules-12-00549-s001.zip › biomolecules-1613564-supplementary.pdf]

Supplementary Information

Investigation of the molecular mechanisms of the eukaryotic cytochrome c maturation System

Silva, A.V.[1]; Firmino, M.O.[1]; Costa, N.L.[1]; Louro, R.O.[1]; Paquete, C.M.[1,*]

Table S1. List of primers used in the study and their purpose.

Nº	Primer	Sequence (5' – 3')	Purpose
1	Histag_FW	CGTCTGTTCTGAAGAAGCACCACCACCACCACCACTAACCATGGGCGAC GTGGAAAAGG	Histag insertion
2	Histag_RV	CTTCTTCAGAACAGACGCAGAAGTACGGCCATCGTCATGGC	
3	ΔCycI_FW	CTTCATCATGAAAGGCTCGCAGGGCCACACGGTGG	CycI removal
4	ΔCycI_RV	CCACCGTGTGGCCCTGCGAGCCTTTCATGATGAAG	
5	H1_FW	CGGATCAAAAATTAGCAGGCTTCCATCTTTTCTCAGGTGGTTGTGAGAG	Insertion of recognition sequence in Heme 1
6	H1_RV	CTCTCACAACCACCTGAGAAAAGATGGAAGCCTGCTAATTTTTGATCCG	
7	H2_FW	GTACCCCTTCTGCTGGCGGTGCCTTATTTTTTGCACAATGCCAA	Insertion of recognition sequence in Heme 2
8	H2_RV	TTGGCATTGTGCAAAAAATAAGGCACCGCCAGCAGAAGGGGTAC	
9	H4_FW	GCCATGCGGTACATGCTGGGAATGTACTCTTTAAACCTACTTGTG	Insertion of recognition sequence in Heme 4
10	H4_RV	CACAAGTAGGTTTAAAGAGTACATTCCCAGCATGTACCGCATGGC	

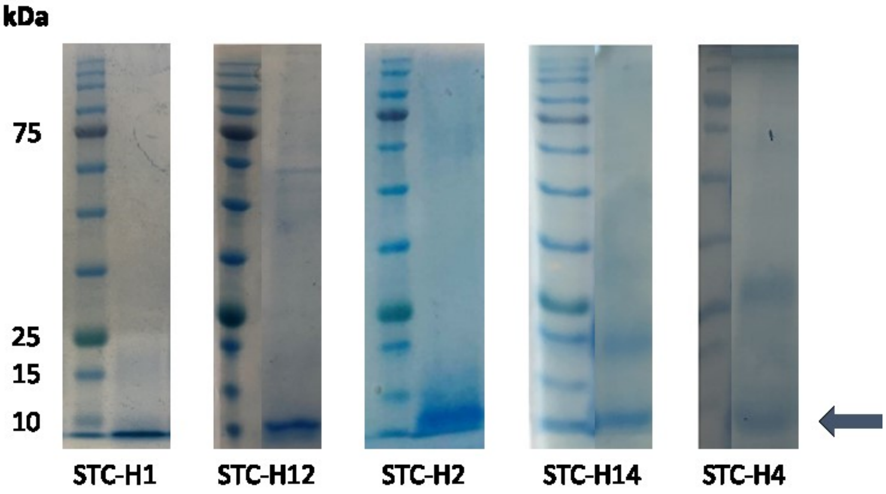

Figure S1. Bluesafe SDS-PAGE gel of STC mutants produced by System III

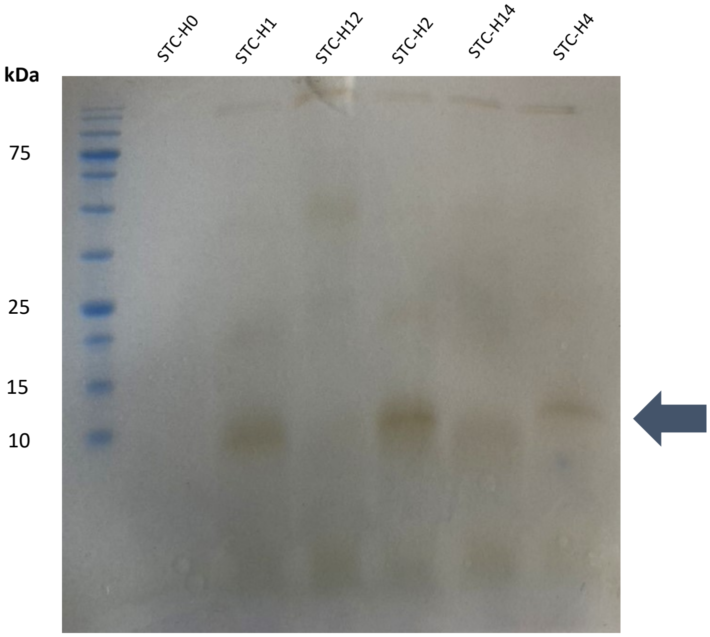

Figure S2. Heme-stained SDS-PAGE gel of STC mutants produced by System III

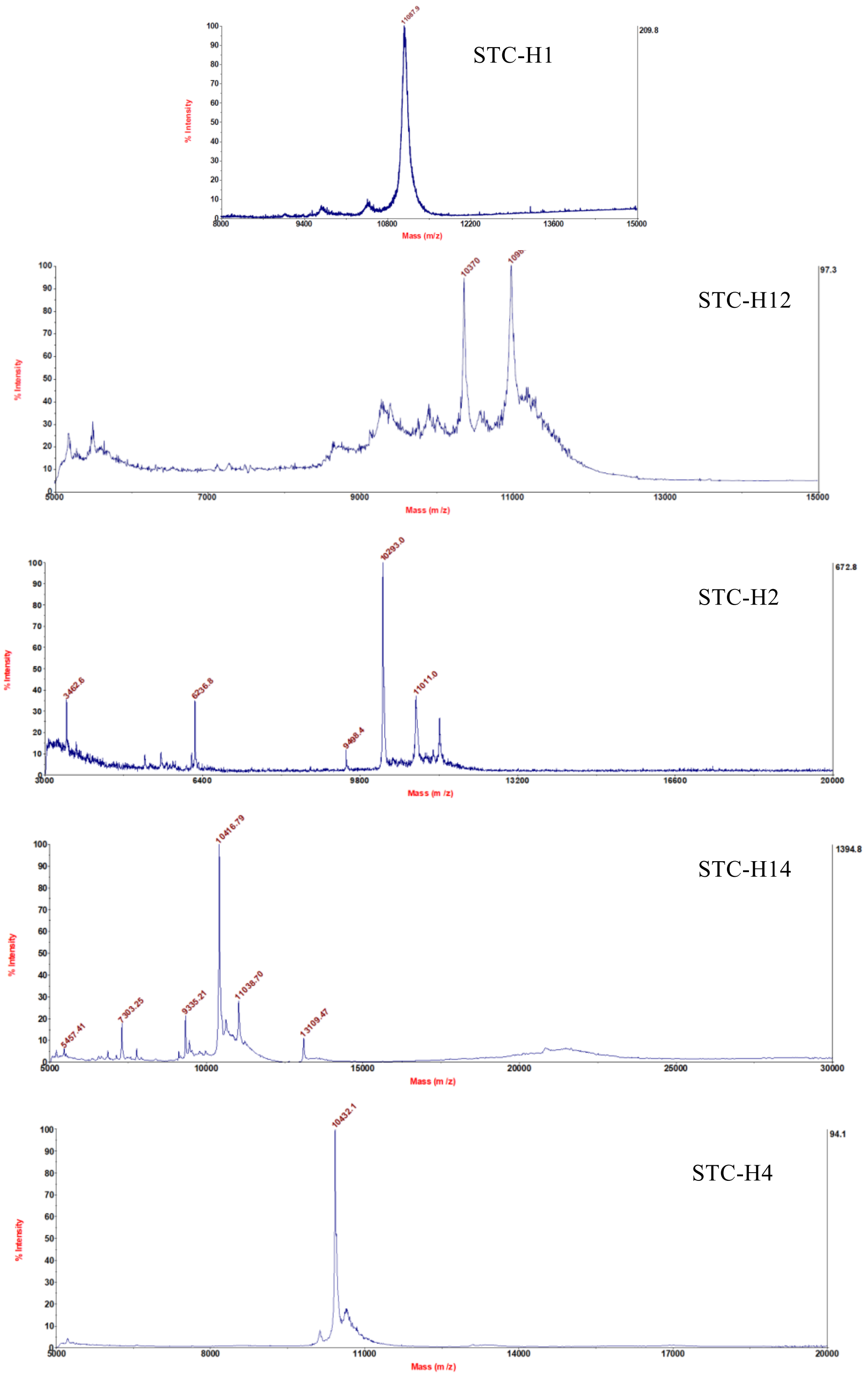

Figure S3. Mass spectrometry of the STC mutants produced by System III in *E. coli*. Data obtained by the Mass Spectrometry Unit (UniMS), ITQB/iBET, Oeiras, Portugal.

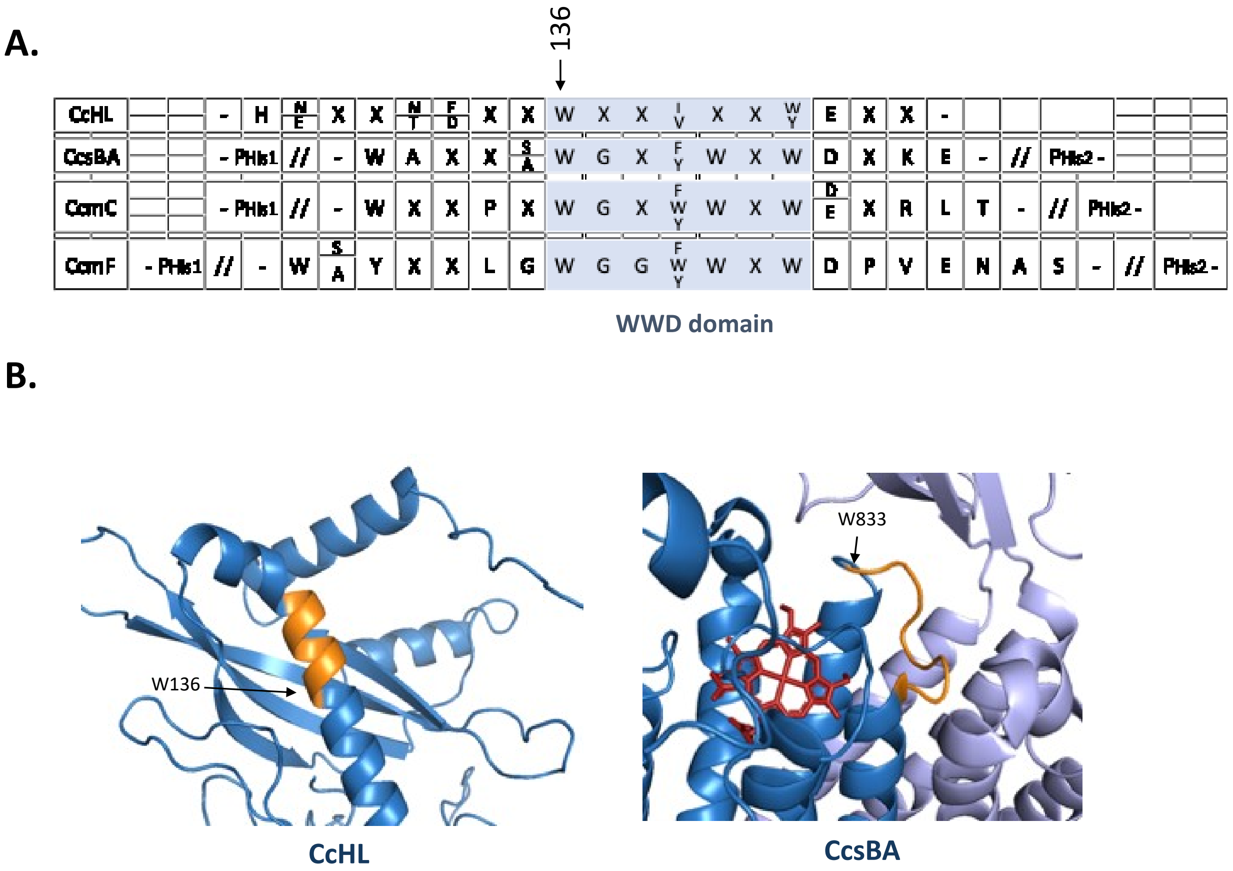

Figure S4. A. WWD domain in the cytochrome maturation Systems I (CcmC and CcmF), II (CcsBA) and III (CcHL), proposed to be homologous and important to recognise the heme binding region and insert the heme in the apo-protein (Babbitt et al. 2015; Mendez et al. 2022). **B.** Cartoon representation of the active site of CcHL (predicted by alpha-fold: AF-P06182-F1) and CcsBA (PDB: 7S9Y) composed by the WWD domain.
